# Optimization of rain gauge sampling density for river discharge prediction using Bayesian calibration

**DOI:** 10.7717/peerj.9558

**Published:** 2020-07-30

**Authors:** Alexandre M.J.-C. Wadoux, Gerard B.M. Heuvelink, Remko Uijlenhoet, Sytze de Bruin

**Affiliations:** 1 Soil Geography and Landscape group, Wageningen University and Research, Wageningen, the Netherlands; 2 Hydrology and Quantitative Water Management group, Wageningen University and Research, Wageningen, the Netherlands; 3 Laboratory of Geo-Information Science and Remote Sensing, Wageningen University and Research, Wageningen, the Netherlands; 4 Current affiliation: Sydney Institute of Agriculture & School of Life and Environmental Sciences, The University of Sydney, Australia

**Keywords:** Sampling design, Bayesian uncertainty analysis, HBV model, Kriging, Inputuncertainty

## Abstract

River discharges are often predicted based on a calibrated rainfall-runoff model. The major sources of uncertainty, namely input, parameter and model structural uncertainty must all be taken into account to obtain realistic estimates of the accuracy of discharge predictions. Over the past years, Bayesian calibration has emerged as a suitable method for quantifying uncertainty in model parameters and model structure, where the latter is usually modelled by an additive or multiplicative stochastic term. Recently, much work has also been done to include input uncertainty in the Bayesian framework. However, the use of geostatistical methods for characterizing the prior distribution of the catchment rainfall is underexplored, particularly in combination with assessments of the influence of increasing or decreasing rain gauge network density on discharge prediction accuracy. In this article we integrate geostatistics and Bayesian calibration to analyze the effect of rain gauge density on river discharge prediction accuracy. We calibrated the HBV hydrological model while accounting for input, initial state, model parameter and model structural uncertainty, and also taking uncertainties in the discharge measurements into account. Results for the Thur basin in Switzerland showed that model parameter uncertainty was the main contributor to the joint posterior uncertainty. We also showed that a low rain gauge density is enough for the Bayesian calibration, and that increasing the number of rain gauges improved model prediction until reaching a density of one gauge per 340 km^2^. While the optimal rain gauge density is case-study specific, we make recommendations on how to handle input uncertainty in Bayesian calibration for river discharge prediction and present the methodology that may be used to carry out such experiments.

## Introduction

Uncertainty analysis has garnered considerable attention in hydrological modelling during the past decades (e.g., Pappenberger & Beven, 2006; Han & Coulibaly, 2017). There is agreement on the necessity to provide (realistic) uncertainty bounds to end-users and practitioners (Beven, 2006; Andréassian et al., 2007; Bal et al., 2014). Brown & Heuvelink (2005) defines uncertainty as an expression of confidence about how well we know the “truth”. Similarly, Maskey (2004) defines uncertainty as a measure of the information about an unknown quantity to be measured or a situation to be forecast, and discusses the nature of different potential sources of uncertainty and their effect on flood forecasting.

It is generally recognized that three principal sources of uncertainty cause uncertainty in model output: model input uncertainty (including initial state and boundary conditions), model parameter uncertainty and model structural uncertainty (Refsgaard et al., 2007; Van der Keur et al., 2008; Houska et al., 2015). For example, Højberg & Refsgaard (2005) found that parameter uncertainty cannot fully cover model structural uncertainty, while Tian, Booij & Xu (2014) showed that parameter uncertainty for three rainfall-runoff models tested on two catchments has a larger contribution to model output uncertainty than model structural uncertainty. Kavetski, Kuczera & Franks (2006) found that input (rainfall) uncertainty has a considerable effect on the predicted outflow and output prediction intervals. In addition to these three main sources, there is usually also uncertainty in the measurements of the model output (Di Baldassarre & Montanari, 2009). This source of uncertainty must be taken into account if these measurements are used to calibrate the model.

Explicit integration of all sources of uncertainty is not an easy task. This problem has been tackled using approaches such as the pseudo-Bayesian Generalized Likelihood Uncertainty Estimation (GLUE) methodology (Beven et al., 2000), the Integrated Bayesian Uncertainty Estimator (IBUNE) (Ajami, Duan & Sorooshian, 2007) and by using Bayesian Total Error Analysis (BATEA) (Kavetski, Kuczera & Franks, 2006). In general, Bayesian analysis has received wide attention because it provides a comprehensive and general framework to specify uncertainty explicitly using probability distributions. It also fosters easy updating of distributions when additional information comes available. The main steps of a Bayesian uncertainty framework are summarized as follows (Kennedy & O’Hagan, 2001): (1) an explicit probability model is specified for each uncertainty source (input, model parameters, model structure), based on prior information; (2) measurements of the model output are used to update the prior distributions to posterior distributions, typically using Markov chain Monte Carlo techniques; (3) the posterior distributions are used to propagate uncertainty in model input, model parameters and model structure to model output for (future) cases where model output is not observed; (4) results are tested against independent validation data to evaluate whether the assumptions made as part of the procedure are realistic.

Numerous studies on Bayesian uncertainty analysis for distributed and physically based hydrological models have been conducted and published. There is general agreement that rainfall-runoff data are often insufficient for supporting reliable inference for complex models involving many spatially distributed physical catchment processes (Beven, 2006; Renard et al., 2010; Laiolo et al., 2016). Wagener et al. (2001) refers to “non-identifiability” leading to “ill-posed” inference of the parameters, which can be avoided by using simpler (lumped) hydrological models with fewer parameters. Lumped hydrological models consider the quantity of interest (e.g. discharge) to be derived from catchment-averaged inputs (e.g. rainfall, potential evapotranspiration). Model inputs often contain substantial error which affect model output. In general, input uncertainty in lumped models is mainly caused by measurement and interpolation errors. For instance, rainfall measurements obtained using rain gauges are not error-free (Habib, Krajewski & Kruger, 2001), while interpolation error is added when rain gauge measurements at point locations are aggregated to spatial averages as needed in lumped rainfall-runoff models. In the case of rainfall, radar images provide time series of spatial rainfall fields (Ciabatta et al., 2016), thus avoiding interpolation error, but these often suffer from complex spatio-temporal errors which make them inaccurate in some circumstances (Cecinati et al., 2017). Thus, rainfall point observations remain a major source for estimating catchment-average rainfall.

There is a recent trend towards a decrease of hydrometric network density (Mishra & Coulibaly, 2009; Keum & Kaluarachchi, 2015). Yet, the uncertainty in average rainfall strongly depends on rain gauge sampling density (Xu et al., 2013; Terink et al., 2018). Hence, a reduction of the rain gauge density will increase the uncertainty about the discharge predicted by the rainfall-runoff model. Renard et al. (2011) used a geostatistical model to infer the catchment-average rainfall and the associated uncertainty from the rain gauges using block kriging. Next, they used the block kriging conditional distribution as a prior in the Bayesian calibration of a lumped rainfall-runoff model. Taking prior knowledge on input uncertainty into account overcomes ill-posedness and significantly improved the accuracy of the runoff predictions (Renard et al., 2010). Clearly, the higher the rain gauge density the narrower the block kriging prior. Thus, a different sampling density leads to a different prior and posterior and ultimately to a different output uncertainty distribution. To the best of our knowledge, little has been done to investigate the effect of rain gauge density on the model output uncertainty within a Bayesian framework.

The objective of this work is to evaluate the effect of rain gauge sampling density on uncertainty in the output of a lumped rainfall-runoff model. The methodology used relies on geostatistics to quantify prior input uncertainty and on Bayesian calibration for model parameter and model structural uncertainty quantification. We calibrate the lumped HBV model (Lindström et al., 1997) using a Bayesian uncertainty framework that accounts for input, parameter, output observation, initial state and model structural uncertainty. Model residuals comprising model structural uncertainty and discharge error are characterized using a time series model, while Markov chain Monte Carlo methods are used to obtain posteriors of the input, model and initial state parameters. The propagation of uncertainties associated with the model input, model parameters and model structure is then analyzed using regular Monte Carlo methods. Several rain gauge density scenarios were tested, each time recalibrating the model and providing discharge predictions with uncertainty intervals. Rainfall posterior intervals as well as model predictive abilities were assessed and discussed. The approach was tested in a case study using ten-day average rainfall and discharge data of the 1,696 km^2^ Thur basin in Switzerland for the years 2004 to 2011.

## Methods

### Rainfall-runoff model

Consider a hydrological model *H* that predicts river discharge from catchment average rainfall. Let **y** = [*y*_1_*y*_2_…*y*_*T*_]^*T*^ and $\overline{\mathbf{z}}=[{\overline{z}}_{1} {\overline{z}}_{2}\ldots {\overline{z}}_{T}]^{T}$ be time series of measured discharge and (known) catchment average rainfall, respectively. We assume that the relation between **y** and $\overline{\mathbf{z}}$, which is governed by the model *H*, is affected by multiplicative measurement and model structural errors, which after log-transformation gives:

(1) \begin{eqnarray*}\log \nolimits (\mathbf{y})=\log \nolimits (H(\overline{\mathbf{z}},\varphi ))+\varepsilon +\eta \end{eqnarray*}

where *φ* is a vector comprising model parameters and the initial state, ε = [ε_1_ε_2_…ε_*T*_]^*T*^ is log-transformed model structural uncertainty and *η* = [*η*_1_*η*_2_…*η*_*T*_]^*T*^ is log-transformed discharge measurement error. Uncertainty in model input (i.e.  $\overline{\mathbf{z}}$) and model parameters (i.e.  *φ*) will be introduced in the next section. It is common (Weerts & El Serafy, 2006; Rakovec et al., 2015) to assume that the *η*_*t*_ (*t* = 1…*T*) are independent and identically distributed normal variates, with constant mean *μ*_*η*_ and variance ${\sigma }_{\eta }^{2}$.

It is unrealistic to have temporal independence for ε and hence we represent it by a first-order autoregressive model (AR(1)):

(2) \begin{eqnarray*}{\varepsilon }_{t}={\beta }_{0}+{\beta }_{1}\cdot {\varepsilon }_{t-1}+{\delta }_{t}, t=1\ldots T {\varepsilon }_{0}\sim \mathcal{N}({\mu }_{0},{\sigma }_{0}^{2})\end{eqnarray*}

where the *δ*_*t*_ (*t* = 1…*T*) are independent and identically distributed normal variates, with zero mean and constant variance ${\sigma }_{\delta }^{2}$. The parameters that characterize ε are merged into a parameter vector $\theta =\{{\beta }_{0},{\beta }_{1},{\sigma }_{\delta }^{2},{\mu }_{0},{\sigma }_{0}^{2}\}$. In this study, *μ*_0_ and ${\sigma }_{0}^{2}$ are assumed known since their effect is typically negligible after a few time steps. Since model structural uncertainty and discharge measurement error are not physically related, ε and *η* are statistically independent. We further require E[*e*^ε^] = E[*e*^*η*^] = 1 to avoid bias in the multiplicative model structural uncertainty and discharge measurement error term, so that their expectation is forced to 1. This is achieved by setting ${\mu }_{\eta }=- \frac{1}{2} {\sigma }_{\eta }^{2}$ and by imposing that $ \frac{2{\beta }_{0}}{1-{\beta }_{1}} + \frac{{\sigma }_{\delta }^{2}}{1-{\beta }_{1}^{2}} =0$, which implies that ${\beta }_{0}=- \frac{1}{2} \frac{1-{\beta }_{1}}{1-{\beta }_{1}^{2}} {\sigma }_{\delta }^{2}$. Thus, parameters *μ*_*η*_ and *β*_0_ are determined by the other parameters and the set of free parameters of ε is reduced to $\theta =\{{\beta }_{1},{\sigma }_{\delta }^{2},{\mu }_{0},{\sigma }_{0}^{2}\}$.

To simplify notation we define $\mathbf{u}=\log (\mathbf{y})-\log (H(\overline{\mathbf{z}},\varphi ))$ and obtain:

(3) \begin{eqnarray*}\mathbf{u}=\varepsilon +\eta .\end{eqnarray*}

### Bayesian uncertainty framework

Conventional estimation of parameters *φ*, *θ* and ${\sigma }_{\eta }^{2}$ using Bayesian calibration (Beven & Freer, 2001; Kavetski, Kuczera & Franks, 2006) starts by using Bayes’ law to derive that the posterior distribution of the parameters is proportional to the product of the likelihood of the observed residuals **u** and the prior distribution of the parameters:

(4) \begin{eqnarray*}p(\varphi ,\theta ,{\sigma }_{\eta }^{2}{|}\mathbf{u})= \frac{p(\mathbf{u}{|}\varphi ,\theta ,{\sigma }_{\eta }^{2})\cdot p(\varphi ,\theta ,{\sigma }_{\eta }^{2})}{p(\mathbf{u})} \propto p(\mathbf{u}{|}\varphi ,\theta ,{\sigma }_{\eta }^{2})\cdot p(\varphi ,\theta ,{\sigma }_{\eta }^{2})\end{eqnarray*}

where $p(\varphi ,\theta ,{\sigma }_{\eta }^{2})$ is a joint prior for the rainfall-runoff model parameters and the model structural and measurement uncertainty parameters. Since there were no physical reasons to include mutual dependencies, the priors for *φ*, *θ*, and ${\sigma }_{\eta }^{2}$ were treated as statistically independent, i.e.  $p(\varphi ,\theta ,{\sigma }_{\eta }^{2})=p(\varphi )\cdot p(\theta )\cdot p({\sigma }_{\eta }^{2})$.

We extend the conventional approach by considering the case where the model input (i.e. the catchment-average rainfall) is also uncertain. Since model output and input are not independent (as is obvious from Eq. (1)) we have $p(\overline{\mathbf{z}}{|}\mathbf{y})\not = p(\overline{\mathbf{z}})$, which implies that model output observations (or observed residuals) should be used to update the probability distribution of the model input, just as these are used to update the distributions of the parameters of the rainfall-runoff model and the model structural and measurement uncertainty. This can be achieved by adding $\overline{\mathbf{z}}$ to the parameters, so that Eq. (4) is replaced by:

(5) \begin{eqnarray*}p(\varphi ,\theta ,{\sigma }_{\eta }^{2},\overline{\mathbf{z}}{|}\mathbf{u})\propto p(\mathbf{u}{|}\varphi ,\theta ,{\sigma }_{\eta }^{2},\overline{\mathbf{z}})p(\varphi )p(\theta )p({\sigma }_{\eta }^{2})p(\overline{\mathbf{z}}).\end{eqnarray*}

We test three different approaches toward including input rainfall uncertainty by estimating the whole set of parameters twice. In the first approach we calibrate $\overline{\mathbf{z}}$ while in the second and third we ignore that discharge measurements inform catchment-averaged rainfall and exclude $\overline{\mathbf{z}}$ from the Bayesian calibration. More information on the approaches tested is given in the next section. Note that, unconditional to **u**, there are no physical reasons to include mutual dependencies between *φ*, *θ*, ${\sigma }_{\eta }^{2}$ and $\overline{\mathbf{z}}$. The model parameter priors *p*(*φ*) can be derived from expert judgment and may be centered around deterministically calibrated parameter values, while uninformative (i.e. wide) priors are typically chosen for *θ* and ${\sigma }_{\eta }^{2}$. The remaining terms of the right-hand side of Eq. (5) are the likelihood $p(\mathbf{u}{|}\varphi ,\theta ,{\sigma }_{\eta }^{2},\overline{\mathbf{z}})$ and the prior for $\overline{\mathbf{z}}$. We work these out in the next two subsections, starting with the latter.

#### Rainfall prior

The rainfall priors for all time instances were derived using a geostatistical approach. Let *z*_*t*_(*s*) denote the rainfall at location *s* in the catchment $\mathcal{A}$ at time *t* ∈ {1…*T*}. We treat ${z}_{t}=\{{z}_{t}(s){|}s&isin; \mathcal{A}\}$ as a realization of a random field *Z*_*t*_. We further assume that log(*Z*_*t*_) is a stationary normally distributed random field, characterized by a (constant) mean and isotropic variogram *γ*(*h*) (where *h* is geographical distance). Since it is unrealistic to assume that log(*Z*_*t*_) has the same statistical properties for all times *t*, in the case study we classified all times into a finite number of classes that are judged sufficiently homogeneous with respect to rainfall intensity, and assumed constant statistical properties within each class. The rainfall intensity classes are ordered by rainfall intensity level (the first class having the lowest intensity and the final class the highest). The class boundaries are based on equidistant quantiles from the cumulative distribution function of the rainfall intensity. Note that while we include spatial autocorrelation, we ignore temporal autocorrelation. In other words, we assume that the correlation between log(*Z*_*t*_(*s*)) and log(*Z*_*t*+*v*_(*s* + *h*)) is zero if *v* ≠ 0. Ignoring temporal autocorrelation is acceptable if the temporal support of rainfall data is sufficiently large (Cecinati et al., 2017) (in the case study we consider rainfall accumulated over ten days). Estimation of the variogram *γ*(*h*) for each rainfall intensity class may be done using the conventional Methods of Moments estimator and by pooling sample variograms derived for all time instants within the same rainfall intensity class (Muthusamy et al., 2017).

To sample from the distribution of *Z*_*t*_ we use conditional Sequential Gaussian Simulation (cSGS) (Cressie, 2015). For each time instant *t*, we first simulate fields of log-transformed rainfall, conditional to the observations log(*z*_*t*_(*s*_*i*_)), *i* = 1…*n*, where *n* is the number of rain gauge locations. Next we back-transform these fields to the original scale to obtain conditional rainfall simulation fields ${z}_{t}^{l}=\{{z}_{t}^{l}(s){|}s\in \mathcal{A}\},l=1\ldots L$, where *L* is the number of simulated fields. Finally, each simulated field is spatially aggregated to obtain catchment average rainfall simulations:

(6) \begin{eqnarray*}{\overline{z}}_{t}^{l}= \frac{1}{{|}\mathcal{A}{|}} \int \nolimits _{s\in \mathcal{A}}{z}_{t}^{l}(s)\mathrm{d}s.\end{eqnarray*}

In practice, the integral in Eq. (6) is approximated by a summation over a (sufficiently dense) spatial grid. The simulations ${z}_{t}^{l}$ are also generated on this same grid. We used a simulation approach to derive the probability distribution of the catchment average rainfall, because analytical solutions are generally not available.

The set of *L* simulations of catchment average rainfall provides an empirical representation of the prior distribution of ${\overline{z}}_{t}$ for all *t*, which is an accurate approximation of the true prior if *L* is sufficiently large. The empirical prior cumulative distribution of ${\overline{z}}_{t}$ is then given by:

(7) \begin{eqnarray*}{F}_{{\overline{z}}_{t}}(a)= \frac{1}{L} \sum _{l=1}^{L}I({\overline{z}}_{t}^{l}\leq a)\end{eqnarray*}

where *I* is an indicator function equal to 1 if its argument is true and 0 otherwise. The probability density function derived from this empirical rainfall distribution is used to obtain density values for each new jump in the (rainfall) parameter space during MCMC.

#### Likelihood function

Deriving the likelihood $p(\mathbf{u}{|}\varphi ,\theta ,{\sigma }_{\eta }^{2},\overline{\mathbf{z}})$ is a major step in the Bayesian model calibration. For notational convenience we will drop the conditioning information in this subsection and write **u** instead of $\{\mathbf{u}{|}\varphi ,\theta ,{\sigma }_{\eta }^{2},\overline{\mathbf{z}}\}$, but note that because of the conditioning information **u** satisfies Eq. (3), with all parameters of ε and *η* known.

We start by writing the joint distribution of **u** as a product of conditional distributions:

(8) \begin{eqnarray*}p(\mathbf{u})=p({u}_{0})\cdot p({u}_{1}{|}{u}_{0})\cdot p({u}_{2}{|}{u}_{0},{u}_{1})\ldots p({u}_{T}{|}{u}_{0}\ldots {u}_{T-1}).\end{eqnarray*}

Because **u** is the sum of ε and *η* it cannot be written as an AR(1) model and does not satisfy the Markov property (Durbin & Koopman, 2012). The conditional distributions can therefore not be reduced to a simple form. Instead, we use a Kalman filter approach (Kalman et al., 1960) to evaluate the conditional distribution in Eq. (8). Since all stochastic variables involved are normal the conditional distributions are also normal, leaving only their means and variances to be determined. These are derived in a recursive way (*t* = 1…*T*):

(9) \begin{eqnarray*}{\hat {\varepsilon }}_{0}^{+}=\mathrm{E} \left[ {\varepsilon }_{0} \right] ={\mu }_{0},\qquad {\sigma }_{0}^{2+}=\mathrm{var}({\varepsilon }_{0})={\sigma }_{0}^{2}\end{eqnarray*}

(10) \begin{eqnarray*}{\hat {\varepsilon }}_{t}^{-}={\beta }_{0}+{\beta }_{1}{\hat {\varepsilon }}_{t-1}^{+}\end{eqnarray*}

(11) \begin{eqnarray*}{\sigma }_{t}^{2-}={\beta }_{1}^{2}{\sigma }_{t-1}^{2+}+{\sigma }_{\delta }^{2}\end{eqnarray*}

(12) \begin{eqnarray*}{\hat {\varepsilon }}_{t}^{+}={\hat {\varepsilon }}_{t}^{-}+{k}_{t}({u}_{t}-{\hat {\varepsilon }}_{t}^{-})-{\mu }_{\eta }\end{eqnarray*}

(13) \begin{eqnarray*}{\sigma }_{t}^{2+}=(1-{k}_{t}){\sigma }_{t}^{2-}\end{eqnarray*}

(14) \begin{eqnarray*}{k}_{t}= \frac{{\sigma }_{t}^{2-}}{{\sigma }_{t}^{2-}+{\sigma }_{\eta }^{2}} .\end{eqnarray*}

Here, ${\hat {\varepsilon }}_{t}^{-}=E[{\varepsilon }_{t}{|}{u}_{0}\ldots {u}_{t-1}]$ is the time update, ${\hat {\varepsilon }}_{t}^{+}=E[{\varepsilon }_{t}{|}{u}_{0}\ldots {u}_{t}]$ the measurement update and *k*_*t*_ is the Kalman gain. The prediction error variances associated with ${\hat {\varepsilon }}_{t}^{-}$ and ${\hat {\varepsilon }}_{t}^{+}$ are given by ${\sigma }_{t}^{2-}$ and ${\sigma }_{t}^{2+}$, respectively. Obtaining the mean and variance of {*u*_*t*_|*u*_0_…*u*_*t*−1_} is now easy and given by:

(15) \begin{eqnarray*}\mathrm{E} \left[ {u}_{t}{|}{u}_{0}\ldots {u}_{t-1} \right] ={\hat {u}}_{t}={\hat {\varepsilon }}_{t}^{-}+{\mu }_{\eta }\end{eqnarray*}

(16) \begin{eqnarray*}\mathrm{var}({u}_{t}{|}{u}_{0}\ldots {u}_{t-1})={\sigma }_{t}^{2-}+{\sigma }_{\eta }^{2}.\end{eqnarray*}

The log-transformed conditional distribution at time *t* > 0 is thus given by:

(17) \begin{eqnarray*}\log \nolimits (p({u}_{t}{|}{u}_{0},\ldots ,{u}_{t-1}))=- \frac{1}{2} \log \nolimits (2\pi )- \frac{1}{2} \log \nolimits ({\sigma }_{t}^{2-}+{\sigma }_{\eta }^{2})- \frac{1}{2} \left\{ \frac{({u}_{t}-{\hat {\varepsilon }}_{t}^{-}+{\mu }_{\eta })^{2}}{{\sigma }_{t}^{2-}+{\sigma }_{\eta }^{2}} \right\} \end{eqnarray*}

while for *t* = 0 we have ${u}_{0}\sim \mathcal{N}({\mu }_{0}+{\mu }_{\eta },{\sigma }_{0}^{2}+{\sigma }_{\eta }^{2})$. Taking the logarithm of Eq. (8) implies that we must sum over all time steps so that the log-likelihood is given by (defining ${\hat {\mu }}_{0}={\mu }_{0}$ and ${\sigma }_{0}^{2-}={\sigma }_{0}^{2}$):

(18) \begin{eqnarray*}\log \nolimits (p(\mathbf{u}))=- \frac{T+1}{2} \log \nolimits (2\pi )- \frac{1}{2} \sum _{t=0}^{T} \left( \log \nolimits ({\sigma }_{t}^{2-}+{\sigma }_{\eta }^{2}) \right) - \frac{1}{2} \sum _{t=0}^{T} \left( \frac{({u}_{t}-{\hat {\varepsilon }}_{t}^{-}+{\mu }_{\eta })^{2}}{{\sigma }_{t}^{2-}+{\sigma }_{\eta }^{2}} \right) .\end{eqnarray*}

#### Markov chain Monte Carlo

The proportionality sign in Eq. (5) means that the posterior on its left-hand side differs from the right-hand side by a multiplicative constant. This constant is unknown (that is to say, it can only be obtained using formidable computer power) and hence the posterior cannot be determined explicitly. To overcome this problem a common approach is to sample from the posterior distribution $p(\varphi ,\theta ,{\sigma }_{\eta }^{2},\overline{\mathbf{z}}{|}\mathbf{u})$ using Markov chain Monte Carlo (MCMC). In this paper we use the Metropolis algorithm, which may not be the most efficient approach but perfectly valid and relatively easy to implement. It is described in detail in Brooks et al. (2011). Thus, a large sample *N* of the joint posterior distribution of $(\varphi ,\theta ,{\sigma }_{\eta }^{2},\overline{\mathbf{z}})$ is generated, where *N* is typically taken in the range 10^4^ to 10^5^. Convergence can be assessed by running several independent Markov chains and checking that the sample distributions are sufficiently similar (Gelman et al., 2014). Another important performance indicator is the acceptance rate, i.e. the number of accepted proposals divided by the total number of proposals. We manually tuned the size of the jump in the parameter space to obtain an acceptance rate between 0.25 and 0.5 (Rosenthal et al., 2011) to obtain sufficient exploration of the parameter space without grossly deteriorating efficiency. The acceptance rate was calculated after removing the first set of proposals, also called the burn-in phase.

### Prediction

Once the joint conditional posterior distribution of all parameters and the catchment-average rainfall (Eq. (5)) has been obtained, it can be used for discharge prediction. To derive the prediction, it is important to distinguish between the true discharge **d** and the measured discharge **y**. From the model defined in Eq. (1) it follows that the log-transformed true discharge log(**d**) = log(**y**) − *η* is the sum of the log-transformed model output $(H(\overline{\mathbf{z}},\varphi ))$ and the log-transformed model structural uncertainty ε. Using the law of total probability, the probability distribution of the discharge can be written as:

(19) \begin{eqnarray*}p(\mathbf{d})=\iiint p(\mathbf{d}{|}\varphi ,\theta ,\overline{\mathbf{z}})\cdot p(\varphi ,\theta ,\overline{\mathbf{z}})\mathrm{d}\varphi \mathrm{d}\theta \mathrm{d}\overline{\mathbf{z}}.\end{eqnarray*}

This multi-dimensional integral is usually solved numerically using Monte Carlo sampling. Since $\log (\mathbf{d})=\log (H(\overline{\mathbf{z}},\varphi ))+\varepsilon $, sampling from $p(\mathbf{d}{|}\varphi ,\theta ,\overline{\mathbf{z}})$ involves a deterministic run of the rainfall-runoff model and simulating a realization from the AR(1) model of ε. One might think that realizations of the posterior $p(\varphi ,\theta ,\overline{\mathbf{z}})$ are already available from the MCMC sampling, but this is not the case. The problem is that realizations of this posterior are only available for the calibration period, i.e. a time period with discharge measurements. Since prediction is only needed for time periods without discharge measurements, we consider a case in which there are no discharge measurements at or near the prediction time. Hence, there are no realizations of the posterior $p(\varphi ,\theta ,\overline{\mathbf{z}})$ for a time period without discharge measurements either. To make the distinction between the calibration and prediction periods explicit, let the prediction period be from *t* = *T* + *V* + 1 to *t* = *T* + *V* + *W*, where *V* is typically larger than the catchment response time to ensure that the validation data are independent from the calibration data. We denote the catchment-average rainfall for the prediction period by ${\overline{\mathbf{z}}}_{+}$. Thus, we derive *p*(**d**_+_) from the (posterior) distribution of *φ*, *θ* and ${\overline{\mathbf{z}}}_{+}$ using Eq. (19), with **d** replaced by **d**_+_ and $\overline{\mathbf{z}}$ replaced by ${\overline{\mathbf{z}}}_{+}$.

We consider three approaches to derive a “posterior” distribution $p(\varphi ,\theta ,{\overline{\mathbf{z}}}_{+})$:

1. For *φ* and *θ*, use the MCMC sample of their joint posterior as explained in the Section on MCMC. For ${\overline{\mathbf{z}}}_{+}$, apply a linear correction to its prior mean as follows:
  (20) \begin{eqnarray*}{\mu }_{{\overline{\mathbf{z}}}_{+}^{po}}=a{\mu }_{{\overline{\mathbf{z}}}_{+}^{pr}}+b\end{eqnarray*}
  where the coefficients *a* and *b* are derived by fitting a linear regression between the means of the rainfall posterior distribution ${\mu }_{{\overline{\mathbf{z}}}_{po}}$ and the means of the rainfall prior distribution ${\mu }_{{\overline{\mathbf{z}}}_{pr}}$ for the calibration period. A similar approach is used in Huard & Mailhot (2008). Thus, realizations ${\overline{z}}_{t}^{l}$ of the rainfall prior are converted to realizations ${\tilde {\overline{z}}}_{t}^{l}$ of the “posterior” by shifting the means, while keeping the same shape and standard deviation:
  (21) \begin{eqnarray*}{\tilde {\overline{z}}}_{t}^{l}={\overline{z}}_{t}^{l}-{\mu }_{{\overline{z}}_{pr}}+{\mu }_{{\overline{z}}_{po}}={\overline{z}}_{t}^{l}+(a-1){\mu }_{{\overline{z}}_{pr}}+b.\end{eqnarray*}
  This approach has the disadvantage that only the mean is corrected, using a simple linear transform. It is not obvious how the correction can be improved. Another disadvantage is that the posteriors of *φ* and *θ* are decoupled from that of ${\overline{\mathbf{z}}}_{+}$. In other words, they are made statistically independent.
2. For *φ* and *θ*, use the MCMC sample from their joint posterior as explained in the section on MCMC. For ${\overline{\mathbf{z}}}_{+}$, let the posterior distribution be identical to its prior. This approach has the disadvantage that the posteriors of *φ* and *θ* are again decoupled from that of ${\overline{\mathbf{z}}}_{+}$.
3. Ignore that discharge measurements inform catchment-averaged rainfall and exclude $\overline{\mathbf{z}}$ from the Bayesian calibration. Thus, the prior distribution of ${\overline{\mathbf{z}}}_{+}$ as derived using block kriging is used as in Approach 2, but unlike in Approach 2 the parameters *φ* and *θ* are calibrated without including $\overline{\mathbf{z}}$ in the calibration procedure. The disadvantage of this approach is that it ignores that rainfall and discharge are dependent, but it has two important advantages. First, there is no interference between calibration of model parameters and rainfall input, which causes problems in the prediction period. Second, the overall number of parameters to be calibrated is much smaller compared to the case in which rainfall input is to be calibrated as well. While this approach essentially boils down to Eq. (4), rainfall uncertainty during the calibration period must be taken into account. This is achieved by integrating the likelihood in Eq. (4) over all realizations of the rainfall $\overline{\mathbf{z}}$:
  (22) \begin{eqnarray*}p(\mathbf{u}{|}\varphi ,\theta ,{\sigma }_{\eta }^{2})=\int \nolimits p(\mathbf{u},\overline{\mathbf{z}}{|}\varphi ,\theta ,{\sigma }_{\eta }^{2})\,\mathrm{d}\overline{\mathbf{z}}=\int \nolimits p(\mathbf{u}{|}\varphi ,\theta ,{\sigma }_{\eta }^{2},\overline{\mathbf{z}})\cdot p(\overline{\mathbf{z}})\,\mathrm{d}\overline{\mathbf{z}}.\end{eqnarray*}
  Note that here we used the fact that $\overline{\mathbf{z}}$ is independent of the model parameters.

The three approaches are different in the way they account for input rainfall uncertainty and in the resultant computational complexity. Approach 1 is the most rigorous way to include input uncertainty in a Bayesian uncertainty analysis. It has the largest flexibility but involves calibration of a rainfall parameter (the catchment average rainfall) for each time step of the calibration period. This increases dramatically the overall number of calibration parameters and the Bayesian uncertainty analysis becomes quickly intractable when the temporal resolution is high. A second problem arises during prediction because in this approach it is not possible to update the rainfall prior derived from geostatistical analysis of the rainfall. This problem may be addressed by using a linear correction (described above), or more simply to use Approach 2 and not correct the prior distribution of the rainfall during prediction. Going one step further, Approach 3 tests if the computational burden of calibrating the rainfall is necessary and whether propagating the input uncertainty can be done by simply sampling from the empirical distribution of the rainfall uncertainty. Approach 3 is the most straightforward way to include rainfall uncertainty into the Bayesian uncertainty analysis.

### Validation measures

To evaluate the methodology it is necessary to statistically validate the discharge predictions and associated prediction uncertainty using independent discharge measurements. We do so for the prediction period, since discharge measurements during this period were not used for calibration and prediction. However, since discharge measurements are not error-free, the validation must take discharge measurement error into account. Discharge measurement error was defined by η in the Methods section. The distribution of η is characterized by a single parameter ${\sigma }_{\eta }^{2}$, the calibration of which was explained in the section on MCMC.

With little modification, prediction equation Eq. (19) can be rewritten to include discharge measurement error:

(23) \begin{eqnarray*}p({\mathbf{y}}_{+})={}p({\mathbf{y}}_{+}{|}\varphi ,\theta ,{\overline{\mathbf{z}}}_{+},{\sigma }_{\eta }^{2})\cdot p(\varphi ,\theta ,{\overline{\mathbf{z}}}_{+},{\sigma }_{\eta }^{2})\,\mathrm{d}\varphi \mathrm{d}\theta \mathrm{d}{\overline{\mathbf{z}}}_{+}\mathrm{d}{\sigma }_{\eta }^{2}.\end{eqnarray*}

Here, **y**_+_ = [*y*_*T*+*V*+1_, *y*_*T*+*V*+2_, …, *y*_*T*+*V*+*W*_]^*T*^ denotes the modelled discharge measurements for the prediction period. The predictions and prediction intervals of **y**_+_ can now be compared to the actual discharge observations to assess the quality of the model. To distinguish between modelled and observed discharge measurements, in this section we denote the first by ${\mathbf{y}}_{+}^{m}$ and the second by ${\mathbf{y}}_{+}^{o}$.

Several measures are employed to assess the quality of the model and the effect of the rain gauge density on the discharge prediction intervals. These are the Mean Error (ME), the Root Mean Squared Error (RMSE), the Nash-Sutcliffe model efficiency coefficient (NSE) and the Prediction Intervals Coverage Probability (PICP). The PICP is the percentage of observations covered by a defined prediction interval (Shrestha & Solomatine, 2008).

(24) \begin{eqnarray*}\mathrm{ME}= \frac{1}{W} \sum _{t=T+V+1}^{T+V+W}({\overline{y}}_{t}^{m}-{y}_{t}^{o}),\end{eqnarray*}

where ${\overline{y}}_{t}^{m}$ is the arithmetic mean of the simulated discharges at time *t*.

(25) \begin{eqnarray*}\mathrm{RMSE}=\sqrt{ \frac{1}{W} \sum _{t=T+V+1}^{T+V+W}({\overline{y}}_{t}^{m}-{y}_{t}^{o})^{2}},\end{eqnarray*}

(26) \begin{eqnarray*}\mathrm{NSE}=1- \frac{\sum _{t=T+V+1}^{T+V+W}({\overline{y}}_{t}^{m}-{y}_{t}^{o})^{2}}{\sum _{t=T+V+1}^{T+V+W}({y}_{t}^{o}-{\overline{y}}^{o})^{2}} ,\end{eqnarray*}

where ${\overline{y}}^{o}= \frac{1}{W} {\mathop{\sum }\nolimits }_{t=T+V+1}^{T+V+W}{y}_{t}^{o}$.

(27) \begin{eqnarray*}\mathrm{PICP}= \frac{100}{W} \sum _{t=T+V+1}^{T+V+W}{I}_{t}\end{eqnarray*}

with

(28) \begin{eqnarray*}{I}_{t}= \left\{ \begin{array}{@{}ll@{}} \displaystyle 1 &\displaystyle \text{if} {y}_{t}^{m}(\mathrm{low})\leq {y}_{t}^{o}\leq {y}_{t}^{m}(\mathrm{up})\\ \displaystyle 0 &\displaystyle \text{otherwise} \end{array} \right. \end{eqnarray*}

where ${y}_{t}^{m}(\mathrm{low})$ and ${y}_{t}^{m}(\mathrm{up})$ are the lower and upper limits of the prediction interval for *y*_*t*_ as computed by the model. In the case study, we will compute the PICP both for the 50% and 90% prediction intervals.

### Sampling density scenarios

To investigate the effect of rain gauge density on the uncertainty of the discharge predictions, several scenarios were developed. Each scenario comprises a number of rain gauges that are optimally selected from the existing rain gauge locations in the study area. The optimal locations were derived using Spatial Simulated Annealing (SSA), by minimizing the time-averaged block kriging prediction error variance:

(29) \begin{eqnarray*} \frac{1}{T+W} \left\{ \sum _{t=1}^{T}{\sigma }_{OK}^{2}(\mathcal{A},t)+\sum _{t=T+V+1}^{T+V+W}{\sigma }_{OK}^{2}(\mathcal{A},t) \right\} \end{eqnarray*}

where ${\sigma }_{OK}^{2}(\mathcal{A},t)$ is the rainfall ordinary block-kriging variance at time *t* and using the entire study area $\mathcal{A}$ as a “block”. Because there is no obvious analytical means to compute the block kriging variance of a lognormally distributed variable, the block kriging variance was approximated by computing the variance of 200 rainfall fields simulated using cSGS, in a similar way as described before. For more details about SSA we refer to Van Groenigen & Stein (1998) and Wadoux et al. (2017).

## Data and model

### Study area

**Figure 1:**
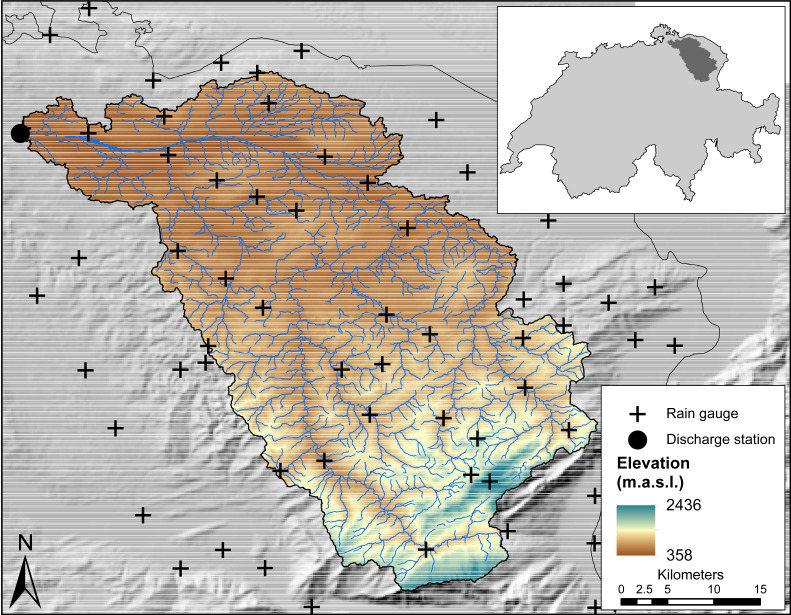
Map of the Thur River Basin with locations of rain gauges and discharge station.

The study area is the Thur basin (1,696 km^2^), located in the North-East of Switzerland (Fig. 1). The Thur river is a tributary of the Rhine river and is the largest non-regulated river in Switzerland. The elevation within the basin ranges from 357 to 2437 m above sea level (m.a.s.l.) with an average height of 765 m.a.s.l. The climate is submontane, relatively cool and dominated by high precipitation, most of which falls during the summer months (June-August) in the form of rain, whereas the winter months (December-February) are characterized by snow. Precipitation is mainly orographic-induced but convective rainfall events also occur in the summer months. The Thur basin has been subject of several previous studies (e.g. Melsen et al., 2016) and data availability is large. Three datasets are used in this study:

• Daily average temperature data for the period 2004-2011 from the Swiss Federal Office for Meteorology and Climatology (MeteoSwiss). Daily temperature is provided as a spatial grid of about 2,300 m × 2,300 m resolution based on an interpolation between meteorological stations (Frei, 2014).
• Daily tipping bucket rain gauge data from MeteoSwiss. Combining manual and automatic gauges, a total of 29 rain gauges also measuring snowfall are available for the period 2004-2011. For the purpose of this study, we included another set of 40 gauges that are within a maximum distance of 20 km from the basin boundary. The rain gauges are distributed evenly in space, with relatively few gauges at high elevation ranges.
• Daily cumulative discharge data for the period 2004-2011 from the Swiss Federal Office for the Environment (FOEN). The discharge measuring station is located at the outlet of the basin at Andelfingen, at an altitude of 356 m.a.s.l.

Meteorological data can be accessed free of charge for teaching and research purposes by request to the IDAweb data internet portal of MeteoSwiss for Science & Research (gate.meteoswiss.ch/idaweb/login.do). Catchment data can be requested at geodata@swisstopo.ch or in the Swisstopo website (swisstopo.ch). Discharge data can be downloaded through the website (bafu.admin.ch) of the Swiss Federal Office of the Environment (FOEN).

### The HBV model

The HBV model (Lindström et al., 1997) is a conceptual lumped rainfall-runoff model developed by the Swedish Meteorological and Hydrological Institute. We chose the HBV model because of its low input data requirement and because it includes a snow melt routine. The required input data consist of time series of catchment-averaged rainfall and air temperature. The model is structured in different routines such as snow melt, evaporation, soil moisture and groundwater. Channel routing is described by a triangular hydrograph. For more detailed information about HBV, we refer to the original publication of Lindström et al. (1997) and to Heistermann & Kneis (2011) for the specific version used in the case study.

### Application to the case study

*Rainfall-runoff model*—We decided to implement a simplified version of the HBV model from the R package RHydro (Reusser, Buytaert & Vitolo, 2017). Time series of rainfall and temperature were split into calibration (2004-2007) and validation (2008-2011) periods. The first year of the two periods (2004 and 2008) was considered as a warm-up period and discarded from the results. The daily time series were aggregated to 10-day averages to keep the number of rainfall parameters that must be calibrated in Approaches 1 and 2 manageable and to avoid convergence problems in the MCMC analysis. The HBV model was run on time steps of 10 days. The impact of the time-steps on the results is discussed more extensively in the Discussion. Prior to the Bayesian calibration and uncertainty analysis, a deterministic calibration was performed. We used a differential evolution algorithm to minimize the Mean Squared Error (MSE) between measured and predicted discharge for the calibration period. The estimated parameters are shown in Table 1 and were used to help define plausible ranges for the priors of the model parameters.

**Table 1:** Model parameters and error model parameters with initial values and prior distributions. The implementation of the HBV model is based on Heistermann & Kneis (2011).

Parameter name	Definition	Initial value through deterministic calibration ^a^	Prior distribution of parameter ^b^
**Model parameters**			
CFMAX	Degree day factor for snow melt [mm/°C/d]	0.324	*Beta*[3, 5, 0, 10]
TT	Temperature threshold below which precipitation falls as snow [°C]	0.387	*Beta*[3, 2, −3, 3]
FC	Field capacity [mm]	106.4	*Beta*[1, 4, 50, 200]
MINSM	Minimum soil moisture for storage [mm]	44.66	*Beta*[1, 4, 0, 200]
BETA	Parameter to control the fraction of rain and snow melt partitioned for groundwater recharge [–]	1.888	*Beta*[1, 1, 1, 5]
LP	Fraction of soil moisture-field capacity-ratio above which actual evapotranspiration equals potential evapotranspiration [–]	0.845	*Beta*[1, 1, 0, 1]
CET	Correction factor for potential evapotranspiration [–]	0.570	*Beta*[1, 1, 0, 20]
KPERC	Percolation coefficient [1/d]	2.670	*Beta*[1, 1, 0, 5]
K0	Fast storage coefficient of soil upper zone [1/d]	0.498	*Beta*[1, 1, 0, 0.5]
UZL	Threshold above which soil upper zone storage empties at rate computed by storage coefficient K0 [mm]	1.226	*Beta*[3, 4, 0, 60]
K1	Slow storage coefficient of soil upper zone [1/d]	0.356	*Beta*[1, 4, 0, 0.5]
K2	Storage coefficient of soil lower zone [1/d]	9.7 × 10^−4^	*Beta*[1, 4, 0, 0.1]
MAXBAS	Length of (triangular) unit hydrograph [d]	3.887	*Beta*[1, 4, 0, 6]
etpmean	Mean evaporation [mm/d]	4.446	*Beta*[1, 3, 0, 50]
tmean	Mean temperature [°C]	4.549	*Beta*[4, 4, −20, 30]
n	(Real) number of storages in linear storage cascade	1.901	*Beta*[1, 2, 0, 7.5]
k	Decay constant for linear storage cascade	1.481	*Beta*[1, 2, 0, 5]
**Initial state parameters**			
snow	Snow storage [mm]	178	*Beta*[1, 3, 0, 200]
sm	Soil moisture storage [mm]	369	*Beta*[1, 3, 0, 200]
suz	Soil upper zone storage [mm]	117	*Beta*[1, 3, 0, 200]
slz	Soil lower zone storage [mm]	44	*Beta*[1, 3, 0, 200]
**Error model parameters**			
*β*_1_	Coefficient for the AR(1) in Eq. (2)	–	*U*[0,1]
${\sigma }_{\delta }^{2}$	Variance for the AR(1), Eq. (2)	–	*U*[0,1]
${\sigma }_{\eta }^{2}$	Measurement error variance, Eq. (1)	–	*U*[0,1]
$\overline{\mathbf{z}}$	Vector of length *T* + *W* for the rainfall parameters	–	${F}_{\overline{\mathbf{z}}}$, see Eq. (7)

**Notes.**

a A deterministic calibration is performed prior to the Bayesian calibration. The parameters are optimized with differential evolution. The objective function is the MSE between the predicted and measured discharge.

b *Beta* [a,b,c,d] represents a beta distribution in the interval [c,d] with shape parameters a and b. *U*[a,b] is a uniform distribution over the interval [a, b].

**Figure 2:**
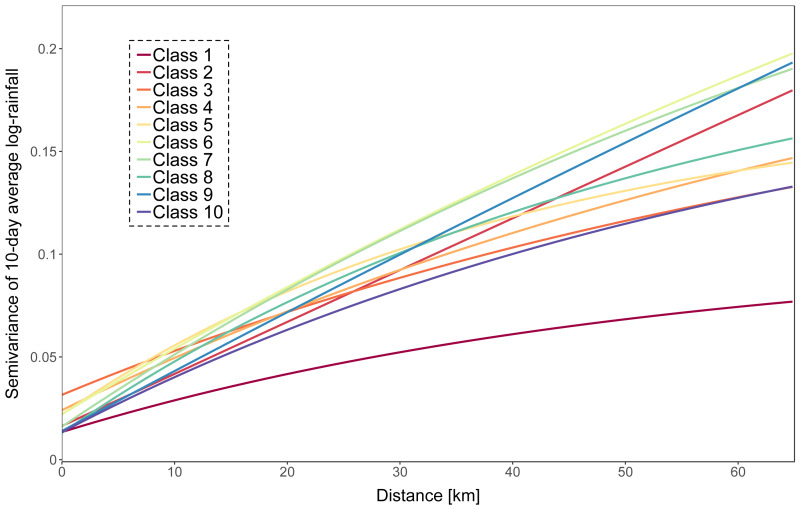
Fitted exponential variograms for each of the ten rainfall classes.

*Rainfall input*—We defined ten rainfall intensity classes based on the 10-day catchment averaged rainfall amounts and fitted exponential variogram models for each class. Rainfall intensity increases with class number. Variogram fitting was based on all rainfall observations that are in the same class, both using rain gauges inside and outside the basin and using an approach described in Muthusamy et al. (2017). A plot of the fitted variograms is presented in Fig. 2. Periods with an average rainfall of less than 0.1 mm were not interpolated and considered as dry. Log rainfall was simulated 500 times on a 1 km × 1 km resolution grid using the class-specific variogram and were conditioned on the rain gauges inside the catchment only. Processing was done using the R package gstat (Gräler, Pebesma & Heuvelink, 2016). Log rainfall simulations were back-transformed at point locations and spatially averaged (Heuvelink & Pebesma, 1999).

Five rain gauge scenarios were considered, comprising 1, 2, 5, 15 and 29 rain gauges, respectively. For each scenario, the rain gauges were selected using SSA by thinning the existing network. The minimization criterion was the average block kriging variance computed by discretization of the area into 500 sub-areas. Implementation was done with the R package spsann (Samuel-Rosa, 2017). The initial SSA temperature was set to 0.1 and the cooling factor was set to 0.8. The total number of SSA iterations was fixed at 10,000. Five out of the eight tested scenarios are reported in this study.

*Bayesian inference*—Model parameters and their priors are shown in Table 1. Prior parameter distributions were chosen based on expert knowledge, previous work in the same basin and optimized parameter values of the deterministic calibration. For each rain gauge scenario the Bayesian inference was performed. The number of MCMC iterations was fixed at 10^6^ for Approaches 1 and 2 and to 10^4^ for Approach 3. The process was repeated several times to ensure convergence of the parameter estimates.

## Results

### Rain gauge optimization

**Figure 3:**
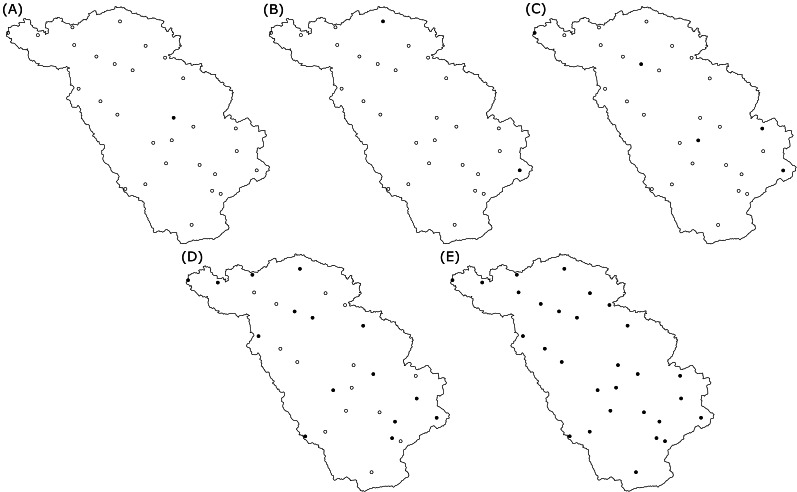
Spatial location of the optimized rain gauges for scenarios of (A) one, (B) two, (C) five, (D) fifteen and (E) all (29) rain gauges. The solid black dots are the selected optimal locations for the rain gauges, empty black dots are the possible candidate locations during the SSA optimization.

Figure 3 shows maps of the study area with the locations of the selected rain gauges, for different rain gauge density scenarios. Recall that during the optimization with SSA, the locations of the rain gauges for a given density are selected from the existing locations. Figure 3 shows that the spread of rain gauges is fairly homogeneous in the geographic space. For the scenario with a single rain gauge, the optimal location is in the center of the study area, while with two rain gauges the optimal positions are near the boundaries. This is a known phenomenon when optimizing sampling locations for mapping with ordinary kriging (Wadoux, Marchant & Lark, 2019).

### Calibration

**Figure 4:**
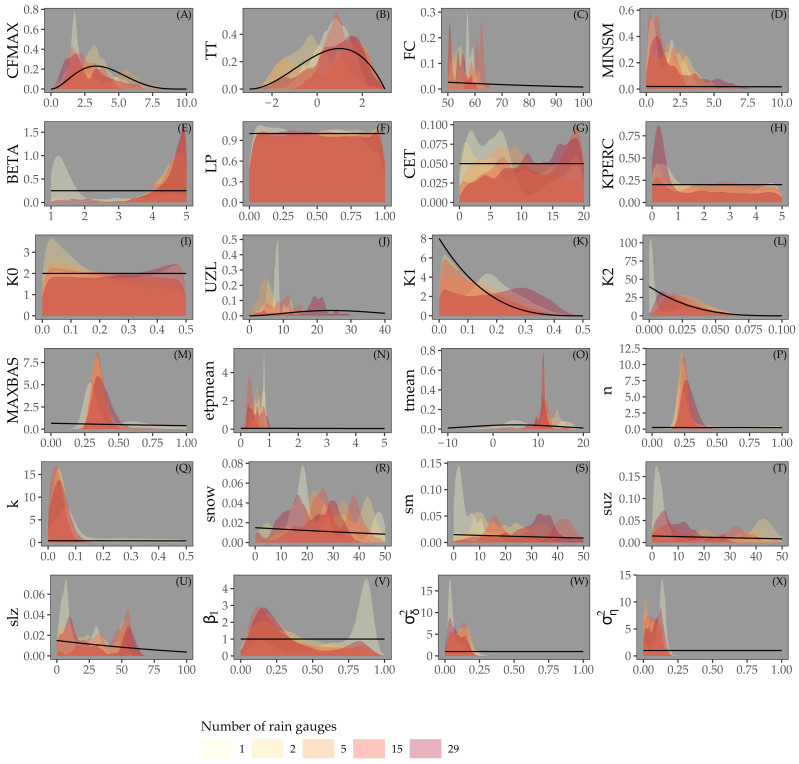
Parameters estimated by Bayesian calibration for Approaches 1 and 2. Black lines in A-X represent prior distributions and colored shapes posterior densities for different rain gauge density scenarios. Rainfall parameter results not shown.

Figure 4 shows the posterior distribution of the calibrated model parameters for Approaches 1 and 2, for different rain gauge density scenarios, along with their prior distribution. For most parameters the posterior distribution is narrower than the prior distribution. This particularly holds for parameters associated with the routing routine (MAXBAS, n, k), the initial state (snow, sm, suz, slz) and the error model (*β*_1_, ${\sigma }_{\delta }^{2}$, ${\sigma }_{\eta }^{2}$). Note that while the posterior distribution of some parameters (e.g. LP, KPERC, K0, MAXBAS and n) is comparable for different rain gauge density scenarios, all other parameter distributions show (large) differences between different rain gauge density scenarios. The distributions do not seem to be narrower in case of higher rain gauge density.

**Figure 5:**
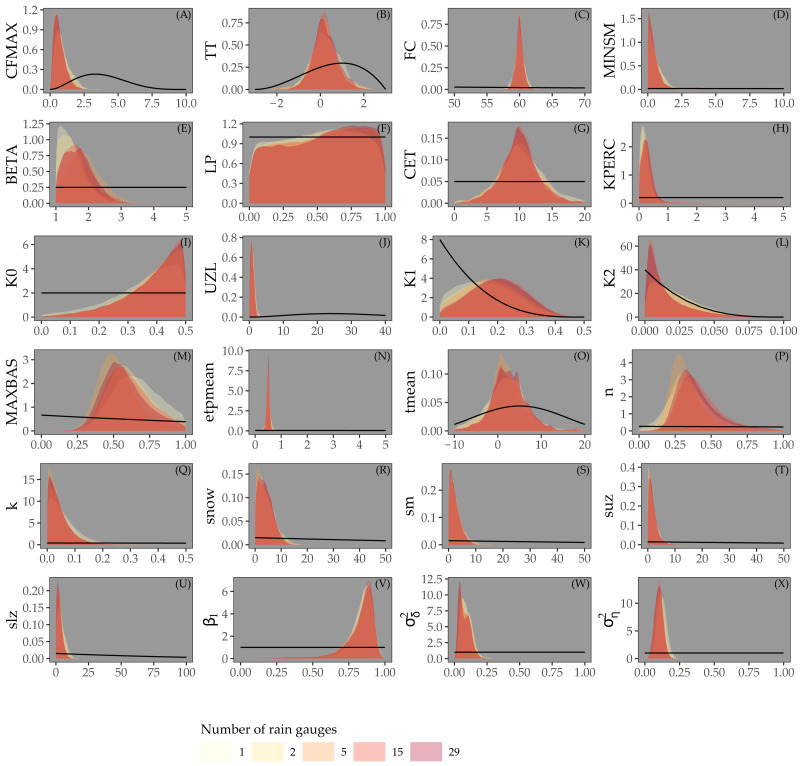
Parameters estimated by Bayesian calibration for Approach 3. Black lines in (A-X) represent prior distributions and colored shapes posterior densities for different rain gauge density scenarios.

Figure 5 shows the prior and posterior distributions of the calibrated parameters for Approach 3, for different rain gauge scenarios. The posterior distributions are much narrower than the prior distributions, particularly for the parameters of the snow routine (CFMAX, TT, FC), the routing routine (MAXBAS, n, k), initial state and error model. For parameters LP, K0 and K1, the posterior distributions are very similar to the priors. In contrast to Fig. 4, all parameters have very similar posterior distributions for different rain gauge densities.

### Prediction

**Figure 6:**
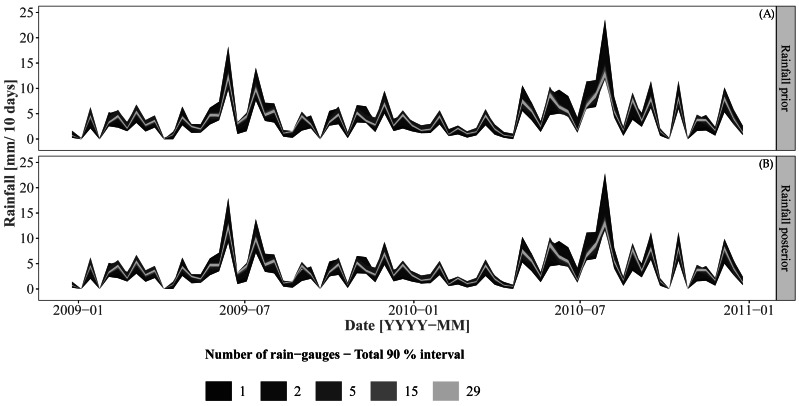
Rainfall priors (A) and posteriors (B) for the prediction/validation period. The rainfall posterior is the corrected prior for Approach 1 for the prediction/validation period. Approaches 2 and 3 sample from the rainfall prior for prediction and validation.

Figure 6 shows the rainfall prior and corrected prior (“posterior”) for the prediction period. Recall that the prior distribution was directly sampled for Approaches 2 and 3 while the rainfall “posterior” distribution was sampled for Approach 1. As expected, the prediction interval width increases when using a smaller number of rain gauges. Both rainfall prior and posterior distributions showed very similar prediction interval widths and mean values. Small differences can be observed when using a small number of rain gauges (i.e. 1 or 2 rain gauges).

**Figure 7:**
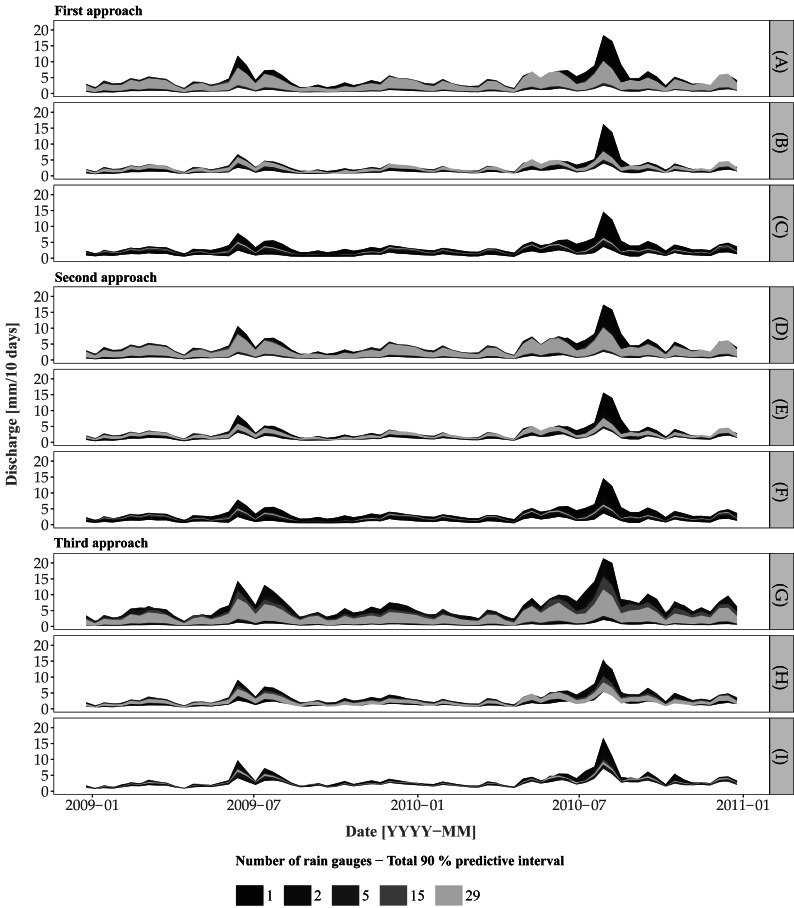
Prediction of the discharge using the three approaches for the cases (A, D, G) where all sources of error are accounted for, (B, E, H) model structural uncertainty is ignored and (C, F, I) model structural uncertainty and model parameter uncertainty (excluding the rainfall input parameters for Approaches 1 and 2) are ignored.

Figure 7 shows the 90% prediction intervals of the discharge for the three approaches and including/excluding various uncertainty sources. For the case where all uncertainty sources are accounted for (plots (A, D. G)), there is a clear pattern towards a smaller width of the prediction intervals with an increase of the number of rain gauges. While there is no clear difference in terms of prediction intervals between the three approaches, Approach 3 provides a larger interval width at a certain time period (e.g., events at 2009-07 and 2010-08) for the scenario involving 15 rain gauges. Note that the differences between rain gauge scenarios are most pronounced for high flows and become negligible for low flows. For example, the scenario involving a single rain gauge shows large uncertainty for all approaches at the discharge peak for the event at 2010–08.

Plots A, D and G in Figures 7A, 7D and 7G do not provide information about the separate effect of input, model parameters and model structural uncertainty on the joint predictive uncertainty. Therefore we also performed an uncertainty propagation analysis that includes/excludes the various uncertainty sources for the cases where model structural uncertainty was ignored and where model structural and model parameter (comprising initial state and error model parameters) uncertainty were both ignored. When model structural uncertainty is ignored (Fig. 7B, 7E and 7H) the uncertainty decreases. With the additional effect of model parameter uncertainty removed (Fig. 7C, 7F and 7I), the prediction intervals become much narrower, with large differences for different rain gauge densities, i.e. the larger the number of rain gauges, the smaller the prediction interval width. The latter reduction is particularly visible when increasing the density from 1 to 5 rain gauges, and becomes marginal when using between 5 to 29 rain gauges. As noted before, the largest difference is obtained for high flow periods.

### Validation

Table 2 shows the validation statistics for the three approaches and the five tested rain gauge scenarios. For the three approaches, increasing the number of rain gauges led to an increase of the predictive power of the model (increase of the NSE). This increase was generally modest, except for Approach 3. It was accompanied by a modest decrease of the residual as characterized by the RMSE. The model predictions were practically unbiased (largest ME deviation from zero equals −0.42), which shows that the prediction inaccuracy was mainly due to random error rather than systematic error. There is no clear pattern regarding the PICP with increasing rain gauge density. For both intervals (50% and 90%), the percentage of observations covered by the interval was within a reasonable range of variation. There is a slight overestimation of the uncertainty for Approaches 1 and 2, while there is a small underestimation of uncertainty for Approach 3 at the 90% interval. Approaches 1 and 2 were very similar in terms of validation statistics, particularly when a large number of rain gauges was used. This is according to expectations, as the effect of the linear correction on the prior diminishes with an increasing number of rain gauges. Approach 2 is more accurate for a small number of rain gauges, while Approach 1 has the largest NSE of all scenarios when using all rain gauges.

## Discussion

### Consistency of parameter estimates

Our experiments suggest that several model parameters estimated in Approaches 1 and 2 might be weakly identified because of their wide posterior distribution. Gelman et al. (2014) stressed that the concept of identification is not so important in the Bayesian perspective and that one must rather look at how much information is supplied by the data, i.e that the joint parameter posterior must occupy less space than the joint prior distribution. In our case, Figs. 4 and 5 show that posteriors were narrower than the priors. This indicates that information was supplied by the data and explains why the parameter posteriors were actually accurate predictors, as shown by the NSE value being higher for Approaches 1 and 2 than for Approach 3, despite the wider posterior distributions of parameters of the third approach.

The posterior of parameter *β*_1_ of the AR(1) model suggests that temporal correlation of the model structural error was weak to moderate, which agrees with findings of Huard & Mailhot (2008). Parameters of the model structural error were well identified. Realistic assumptions regarding the model structural error model formulation play a major role to distinguish between model structural and input uncertainty. Since model structural uncertainty is incorporated explicitly it is unlikely that input uncertainty compensates for deficits in the model structure (Thyer et al., 2009). We followed the approach of Beven & Freer (2001) that model structural uncertainty can be described by a first order autoregressive model. We acknowledge that more complex structures of model residuals can be formulated, such as by using an ARMA or ARIMA model, but this would be at the expense of increasing parameter space dimensionality.

**Table 2:** Validation measures for the three approaches and five rain gauge densities, computed over the period 2009–2011.

	Number of rain gauges				
	1	2	5	15	29
**Approach 1**					
NSE	0.28	0.28	0.48	0.54	0.56
RMSE (mm/10 days)	1.25	1.25	1.06	1.01	0.99
ME (mm/10 days)	−0.42	−0.23	−0.31	−0.15	−0.16
PICP 50%	52.72	58.18	53.63	61.64	59.69
PICP 90%	90.00	90.63	94.54	92.72	94.54
**Approach 2**					
NSE	0.39	0.48	0.45	0.49	0.50
RMSE (mm/10 days)	1.15	1.06	1.09	1.04	1.01
ME (mm/10 days)	−0.19	0.08	−0.02	−0.03	0.09
PICP 50%	54.54	60.00	59.99	62.63	60.00
PICP 90%	94.54	92.72	92.72	93.63	94.54
**Approach 3**					
NSE	0.17	0.24	0.36	0.38	0.42
RMSE (mm/10 days)	1.37	1.27	1.18	1.15	1.12
ME (mm/10 days)	0.28	0.17	−0.11	0.10	0.06
PICP 50%	55.45	56.36	51.81	52.72	53.63
PICP 90%	88.18	86.36	84.54	86.36	85.45

### Prediction

In contrast to many studies reported in the literature (e.g. Kavetski, Kuczera & Franks, 2006), analysis of the predictive uncertainty shows that in the case study the contribution of rainfall uncertainty is relatively small and that discharge predictive uncertainty is mainly dominated by model structural and model parameter uncertainty. As a consequence, the effect of the rain gauge density diminishes if model parameter and model structural uncertainty are accounted for. This was different in the case of using a single rain gauge for calibration. In this case, rainfall uncertainty was an important contributor to the total predictive uncertainty, particularly for the peak flow. Large model parameter and model structural uncertainty offsets small input rainfall uncertainty. In particular for Approach 3, model structural uncertainty is clearly the largest contributor to the total predictive uncertainty. Several investigations obtained similar results. For example, Engeland, Xu & Gottschalk (2005) showed that model structural uncertainty is larger than model parameter uncertainty for simple conceptual models with few well-defined parameters. Our results also confirm the study of Talamba, Parent & Musy (2010) for a lumped hydrological model. Talamba, Parent & Musy (2010) showed, for a fully distributed hydrological model, that accounting for input rainfall uncertainty did not lead to a substantial change in terms of estimated parameters and model performance, because other sources of uncertainty dominated the total predictive uncertainty. This is similar to our case study, where in all three approaches the prediction intervals have a similar width and range. The validation measures show that Approaches 1 and 2, i.e. the case where rainfall parameters are calibrated, outperform Approach 3.

### Differences between the three approaches

Comparison of the three approaches revealed that Approach 3 has larger prediction intervals and poorer model performance, despite the fact that in Approach 3 most parameters have a well-defined unimodal posterior distribution. Note also that the choice of approach leads to different posterior ranges of parameter estimates. The smaller number of parameters to calibrate in Approach 3 (i.e. the input rainfall parameters are not calibrated) suggests that inference benefits from the reduced dimensionality of the parameter space. The validation results show that this was not the case when a small number of rain gauges is used (for instance, NSE = 0.17 using 1 rain gauge). Thyer et al. (2009) and Huard & Mailhot (2008) reported similar results when calibrating time-dependent rainfall input parameters. They showed how calibrating input rainfall parameters for each time step compensates for the situation where a rainfall event is not recorded by a small number of rain gauges, and how this can lead to a near-perfect match between the observed and predicted discharge. In the latter case, Huard & Mailhot (2008) demonstrated that, since model and input rainfall parameters are estimated jointly, it is likely that the input rainfall parameters compensate for structural deficits of the model. In our case this was avoided by: (i) explicitly accounting for model structural uncertainty; and (ii) defining meaningful priors for the input rainfall parameters using geostatistical analysis. Note that these conclusions could be balanced with the use of radar remote sensing images in combination with the rain gauges to estimate the rainfall. Using one single rain gauge could be sufficient, provided that the radar image detected the rainfall event (Di Piazza et al., 2011).

From a numerical perspective, a major difference between Approaches 1 and 2 and Approach 3 is the number of parameters to calibrate. Hydrologists tend to shy-away from high-dimensional rainfall input parameter space, because it often leads to question the statistical significance of the inferred parameters (e.g. Vrugt et al., 2008). A solution is to resort to MCMC search algorithms, which are efficient to explore the multi-dimensional and correlated parameter space. This has been recently tackled in the hydrological literature (e.g. by Laloy & Vrugt, 2012) and in the more general statistical literature (e.g. Ter Braak, 2006). Another solution is to reduce the dimension of the parameter space. Alternative methods to Approach 1 (i.e. the case where each time step is an input rainfall parameter to calibrate) exist. For example, Kavetski, Kuczera & Franks (2006) use storm-event multipliers under the assumption of perfect dependence of input errors within single storm events. By letting these multipliers vary according to the plausible range of hydrological variation, they correct for systematic error in the rainfall input. The major limitation is the need to define hydrological ranges in which the calibrated multiplier is kept constant.

In practical terms, one would not make a serious flaw by taking Approach 3 and not calibrating the input rainfall uncertainty as additional parameters in the Bayesian uncertainty analysis. This conclusion is particularly valid when the number of rain gauges is larger than five in our case study, but this might not hold for all cases. In all approaches, the uncertainty was accurately quantified. This means that despite poorer predictive performance, Approach 3 still yields valuable information because the prediction uncertainty is fairly low. In most cases, practitioners are interested in reliable prediction of the discharge, but given the computational complexity of Approaches 1 and 2, modellers might rightfully opt for Approach 3 with negligible consequences in terms of uncertainty quantification and a relatively small decrease of predictive performance.

### Implications for rain gauge density

The impact of the rain gauge density on parameter posterior distributions was modest. Parameter posteriors for Approaches 1 and 2 show that there was typically little difference between the rain gauge scenarios, while there was almost no difference in the case of Approach 3. A difference is found in Approaches 1 and 2, where using a single rain gauge led to a different posterior distribution of some parameters. This suggests that parameter estimation was robust to the density of the rain gauges, provided that the network is composed of more than a single rain gauge. This is an important finding, as the necessary condition for the regionalization of a rainfall-runoff model is that the parameters are insensitive to the choice of the number and locations of rain gauges (Thyer et al., 2009). We acknowledge that these results may be different in different circumstances, such as in a case where rainfall uncertainty has a larger impact on the parameter posterior distributions, or in the case of using remote sensing images and environmental predictors as covariate in the kriging step (Bostan, Heuvelink & Akyurek, 2012). This study, however, contradicts the findings of Zeng et al. (2018), who found that parameter posterior distributions vary considerably under different rain gauge densities. However, Zeng et al. (2018) did not propagate rainfall input uncertainty and simply sampled a large number of possible rain gauge combination for a given density and analyzed the differences between rain gauge densities in terms of the model parameter posterior distribution. Thus, they ignored a substantial proportion of the uncertainty, which potentially caused model parameter uncertainty to compensate for the unaccounted rainfall uncertainty (Kavetski, Kuczera & Franks, 2006).

In our experiment, low rain gauge densities already produced accurate model predictions. This particularly applied to Approaches 1 and 2 where only five gauges led to a NSE greater than 0.45 (two gauges are sufficient in Approach 2 for a NSE of 0.48). This threshold is low compared to other studies. Dong, Dohmen-Janssen & Booij (2005) reported that five rain gauges were enough to calibrate the HBV model in a 17,000 km^2^ basin in China, using the expected variance of the areal rainfall as a measure of input uncertainty. In a 3,234 km^2^ catchment in France, Anctil et al. (2006) concluded that ten rain gauges (out of 23) is an absolute minimum to predict discharge using a neural network model. However, the rainfall spatial variation was not modeled explicitly in this study. Bárdossy & Das (2008) found that the overall model performance worsened radically with an excessive reduction of rain gauges in the upper Neckar catchment of about 4,000 km^2^. They optimized the rain gauge locations for different rain gauge densities using simulated annealing and kriging with external drift. They showed a significant reduction of the rainfall input variance with increasing density, which paired with a decrease of the discharge prediction error. However, the cited studies did not use Bayesian calibration. In a Bayesian framework, Zeng et al. (2018) found that 10–15 rain gauges were necessary to obtain stable parameter estimates for medium-size sub-basins, but the propagation of the input rainfall uncertainty was not analyzed in this study. In our study, the input rainfall uncertainty was estimated using geostatistics and propagated in a Bayesian framework. A fairly accurate estimate of the catchment average 10-day rainfall was obtained using just two or five rain gauges, which explains why a surprisingly small number of rain gauges was enough to calibrate the hydrological model. The rainfall posterior parameters adjust for the missing information using the discharge data. In Approach 3, i.e. when the rainfall is not updated by the discharge data, the model performance was worse than the other approaches in all cases, and particularly for cases with a small number of rain gauges (fewer than five rain gauges).

Although using a small number of rain gauges led to accurate model prediction, using more rain gauges improves the model predictions. The results of our case study showed that a density larger than five rain gauges led to a marginal improvement of the prediction accuracy. This is equivalent to one rain gauge per 340 km^2^. It should be noted that this result cannot easily be generalized because it is likely case-dependent. In particular, when optimizing the number of rain gauges we assumed that these are placed optimally so that the block kriging variance is minimal, while in practice the gauges might be clustered at some parts (e.g. at lower altitude) of the catchment. In such case, we may need more gauges to reach the same block kriging variance. The surprisingly low rain gauge density is also likely related to the 10-day time step that we used in the case study. Aggregating rainfall over 10-day periods automatically leads to a decrease of the rainfall spatial variation. Figure 2 shows that the spatial variation of the 10-day average rainfall in the Thur basin is relatively small: the variogram sill was small and the variogram range was large. Note also that we assumed a log-normal distribution of the rainfall amount at point support, but that more complex models can be used to account for the typically skew and zero-inflated rainfall distribution (Engeland et al., 2016). We emphasize that predicting 10-day average discharge also leads to smoothing and hence will miss peak discharges. For certain applications a smaller time step will be required, although the time step that we used is suitable for many applications, e.g. for total discharge prediction over long time periods or agricultural water management. Despite data availability, we did not use subcatchments and/or elevation zones but focused on the total river discharge prediction. This study could theoretically be extended to investigate the effect of rain gauge density and rainfall variability on several of the Thur gauged sub-catchments, with a distributed or semi-distributed model. In this way, it would be possible to assess the effect of rainfall spatial distribution on the discharge at the catchment outlet. This would undoubtedly have an impact on the optimal rain gauge sampling design and density, but this approach would be at the expense of numerical efficiency.

### Limitations, extensions and improvements

Finally, we emphasize that in this study we made several assumptions and modelling choices that may not hold for other case studies and/or other model applications.

The log-transformed discharge measurement error was assumed normally distributed and constant over time. This is a common assumption in hydrological modelling (e.g. Huard & Mailhot, 2008). In fact, several authors (e.g. Weerts & El Serafy, 2006; Mazzoleni et al., 2018) model discharge error by a normal distribution with standard deviation fixed at 10% of the measured discharge. In this study we did not fix the measurement error variance but estimated it from the data, together with other model parameters. Further, we avoided the common assumption of temporal independence of log-transformed model structural uncertainty, made for example by Rakovec et al. (2015) and Mazzoleni et al. (2018). We judged that it was unrealistic to assume that deficiencies in model structure are independent in time and therefore accounted for temporal dependence using a first-order auto-regressive model. In future research more elaborate ARIMA models could be used to represent model structural uncertainty.

For several hydrological applications, such as flood forecasting, daily or sub-daily rainfall information is required to capture the high variability of peak flows. The conclusions drawn in this study regarding rain gauge density will likely not hold when rainfall variability increases or rainfall and discharge data are aggregated over smaller time steps. Likely there is also an important effect of catchment size and/or location. The minimum average density of rain gauges required for modelling (in our case study one rain gauge per 340 km^2^) may not hold for smaller catchments or sub-catchments, while it might be a too high density for very large, continental-scale catchments. As mentioned before, the effect of elevation on rainfall and discharge prediction error was not included in this study either, although it may have an impact on the optimal locations of the rain gauges. The use of radar/satellite derived rainfall fields (e.g., Huza et al., 2014; Cecinati et al., 2017) may also have a significant impact on the uncertainty of the rainfall fields and therefore on the joint posterior uncertainty of the discharge. This might be particularly useful if combined with spatially-distributed hydrological models, such as the WaSIM-ETH (Schulla & Jasper, 2007) and wflow_sbm (Imhoff et al., 2020) models. Our methodology also applies to spatially distributed modelling approaches, although computing time will likely increase.

Since our conclusions are case-study specific, this means that for each case study, one should design an experiment to derive the minimum number and locations of rain gauges to be used in hydrological model calibration. In this work we have presented a methodology that may be used to carry out such experiments.

## Conclusion

We calibrated the HBV rainfall-runoff model accounting for input, parameter, initial state and model structural uncertainty using a Bayesian framework for a 1,700 km^2^ basin in Switzerland. Prior input rainfall distributions were derived using a geostatistical approach. We tested several scenarios for incorporating the input uncertainty and assessed the effect of rain gauge density on calibration. The main conclusions are:

• Assumptions regarding the formulation of the model structural uncertainty play a major role in distinguishing between model structural and input uncertainty. Since model structural uncertainty is incorporated explicitly it is unlikely that input uncertainty compensates for deficits in the model structure.
• In our case study, input uncertainty was small compared to model structural and parameter uncertainty.
• Calibrating the rainfall parameters (Approach 1) led to more accurate model performance compared to the case where rainfall uncertainty was not updated using discharge data (Approaches 2 and 3). The increased dimensionality of the parameter space when calibrating rainfall did not lead to computational intractability.
• Parameter estimates were robust to rain gauge density. This is important, as this enables regionalization of the rainfall-runoff model.
• In our case study, using only two rain gauges did not seriously deteriorate discharge prediction.
• Adding up to five rain gauges improved the model prediction. Adding even more only produced a marginal improvement of the prediction accuracy. For our study area, five rain gauges is equivalent to one rain gauge per 340 km^2^.

## Additional Information and Declarations

### Data Availability

The following information was supplied regarding data availability: This work uses publicly available data. Meteorological data can be accessed free of charge for teaching and research purposes upon request to the IDAweb data internet portal of MeteoSwiss for Science & Research (gate.meteoswiss.ch/idaweb/login.do) after registration, and by searching the database name: RdissaggH. Catchment data can be requested at geodata@swisstopo.ch or in the Swisstopo website (swisstopo.ch), accession number: GWN07 of VECTOR25. Discharge data can be downloaded through the website (bafu.admin.ch) of the Swiss Federal Office of the Environment (FOEN), database number 136, station code S1 and FOEN code 2044.
